# Scabies in Infants: Series of 51 Cases

**DOI:** 10.3390/children11040443

**Published:** 2024-04-07

**Authors:** Isabel Betlloch-Mas, Elena Boluda-Verdú, Noelia Jara-Rico, Verónica Sánchez-García, Laura Berbegal-De Gracia, Eusebi Chiner-Vives

**Affiliations:** 1 Department of Clinical Medicine, Miguel Hernández University, Carretera Alicante-Valencia N-332, 03550 Sant Joan d’Alacant, Spain; elena.boluda01@goumh.umh.es; 2 Department of Dermatology, Dr. Balmis General University Hospital, 03010 Alicante, Spain; jara_noe@gva.es (N.J.-R.); sanchez_veronicagar@gva.es (V.S.-G.); berbegal_laudeg@gva.es (L.B.-D.G.); 3 ISABIAL Research Institute, 03010 Alicante, Spain; 4 Department of Pulmonology, San Juan de Alicante University Hospital, 03010 Alicante, Spain; echiner@umh.es

**Keywords:** scabies, infants, childhood scabies

## Abstract

We conducted a two-year retrospective evaluation of infants aged under two years with a confirmed, clinical, or suspected diagnosis of scabies in a healthcare center in Alicante (Spain) to determine possible factors associated with diagnostic delay and poor treatment response. We collected epidemiological, clinical, diagnostic, and treatment variables. After describing our findings as mean values and percentages, we compared categorical variables using the Student’s *t*-test and the Mann–Whitney U test, and we compared continuous variables with the Chi^2^ test and Pearson’s correlation coefficient. We included 51 infants (19 boys and 32 girls) with a mean age of 15 months. The main source of contagion was the family; half of the infants lived with four or more people. According to the International Consensus Criteria for the Diagnosis of Scabies, confirmed scabies was diagnosed in 45% of cases and clinical scabies in 47%, and 45% of cases had a diagnostic delay. Lesions mainly affected the hands, feet, and trunk, with papules in 92% of cases and burrows in 55%. The predominant symptoms were pruritus (94%) and irritability (69%). Regarding treatment, 98% of the infants received topical permethrin and 35% received oral ivermectin. Treatment failed in 76% of infants. Living in large family units was associated with a higher risk of contagion and therapeutic failure. Diagnostic delay was associated with previous misdiagnosis.

## 1. Introduction

Scabies is an infestation of the skin by the ectoparasitic mite *Sarcoptes scabiei* var. *hominis*. It affects an estimated 200 million people worldwide [1,2]. In 2017, the World Health Organization (WHO) included scabies in its list of neglected tropical diseases [3]. Several studies reported a marked increase in scabies cases in Europe [4] and worldwide [5,6,7] during the coronavirus 2019 (COVID-19) pandemic, and another study observed a postpandemic rise in cases due to diagnostic delay [8]. In Spain, the incidence of scabies has increased since 2014, probably owing to social and health cuts during the economic crisis, among other factors [9].

Scabies is currently a global health problem that affects people of all ages, including children [10,11]. The incidence is particularly high in children aged under two years (infants) [3,11,12].

The clinical presentation of scabies varies depending on the age of the affected person. Adults normally have a very itchy rash, with papules, burrows, and vesicles in the web spaces and on the wrists, armpits, genitals, and buttocks; whereas in infants and young children, the lesions are very polymorphous (papules, vesicles, pustules, nodules, and excoriated eczematous areas), and the most commonly affected areas are the head, palms, soles, and ankles [13]. The diagnosis of scabies can be challenging in infants because of their inability to express discomfort and the divergent clinical presentation and the fact that many infants show no signs of pruritus; as a result, diagnosis and resolution may be delayed, facilitating the spread of the infestation [14].

Identifying the clinical and epidemiological characteristics of infants affected by scabies could help to improve diagnosis of this infestation, which disproportionately affects children younger than two years [15].

The aim of this study was to record the epidemiological, clinical, and treatment characteristics of all infants diagnosed with scabies in our hospital over two years and evaluate possible factors associated with diagnostic delay and poor therapeutic response.

## 2. Materials and Methods

We conducted a retrospective study of all children aged under two years diagnosed with scabies in our hospital (Dr. Balmis General University Hospital, Alicante, Spain) from October 2021 to September 2023. We recorded the following data.

- Epidemiological variables: age, sex, number of household members, ethnicity, and source of contagion;
- Clinical variables: type of lesions, location of lesions, symptoms (e.g., pruritus, pain, and fever), and complications;
- Diagnosis and treatment variables: time to diagnosis, prescribed treatments, and time to healing.

Diagnoses were based on criteria established by the International Alliance for Control of Scabies (IACS) in 2020 [16]. We defined diagnostic delay as a period of more than two weeks between the onset of symptoms and diagnosis or initiation of treatment. We considered that treatment had failed when the time from diagnosis/initiation of treatment to resolution (healing of previous lesions and absence of new lesions) was more than 30 days, or where the infant required more than two cycles of permethrin or more than one cycle of oral ivermectin.

For the statistical analysis, we used SPSS version 18.0 (Chicago, IL, USA). For the descriptive study, we expressed continuous variables as means and standard deviations (SDs) and the distribution of frequencies as percentages. To compare categorical variables, we used the Chi^2^ test or Fisher’s exact test. For the numerical variables, we used the Student’s *t*-test or the Mann–Whitney U test. We used Pearson’s or Spearman’s coefficients to correlate numerical variables. To compare continuous variables among more than two groups, we used analysis of variance (ANOVA or the Kruskal–Wallis test). A *p* value below 0.05 was considered significant [17]. The hospital ethics committee approved the study (ethics committee reference: PI2023-148).

## 3. Results

Table 1 shows the epidemiological characteristics of our series. Of 170 children (aged 14 years or younger) diagnosed with scabies in our hospital during the study period, 51 were infants. The mean age of the infants was 15 months (SD 7 months), and 25 infants (49%) were younger than 12 months. Thirty-two infants (63%) were girls. The source of contagion was the family in 48 cases (94%) and daycare in 3 cases (6%).

An epidemiological factor associated with contagion was the mean number of household members, which was six (SD 1). Half of the infants (*n* = 26, 51%) lived in households with five or more members, and six infants (12%) lived with nine or more other people. More than half of cases were concentrated in a single neighborhood. A total of 35.3% of the families were of European descent, while the rest were of Romani, Latin American, or Middle Eastern/North African descent. Other factors, such as prematurity and corticosteroid therapy, were irrelevant in our study, while 27% of the infants had atopic dermatitis.

**Figure 1:**
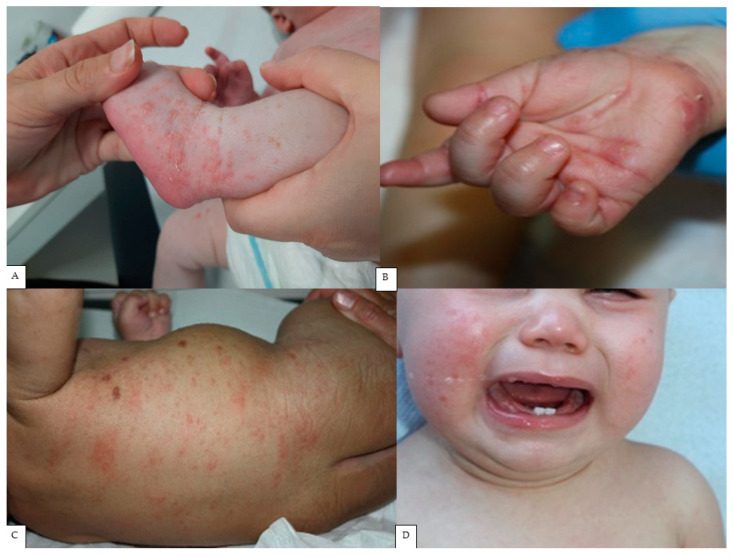
Typical scabies lesions in infants: burrows on the dorsa of the feet (**A**). Pustules and vesicles on the hands (**B**). Papules and nodules on the trunk (**C**). Facial lesions and irritability (**D**).

The predominant lesions were papules (*n* = 47, 92%), excoriation (*n* = 38, 75%), scaly areas (*n* = 36, 71%), pustules (*n* = 29, 57%), and burrows (*n* = 28, 55%), while nodules were observed in 18 cases (35%). The main locations of lesions were the trunk (*n* = 42, 82%), palms/soles (*n* = 32, 63%), arms (*n* = 29, 57%), folds (*n* = 23, 45%), and head (*n* = 20, 38%). The most common symptoms were pruritus (*n* = 48, 94%) and irritability (*n* = 35, 69%). Eight infants (16%) had respiratory symptoms and fever (Table 2; Figure 1).

**Figure 2:**
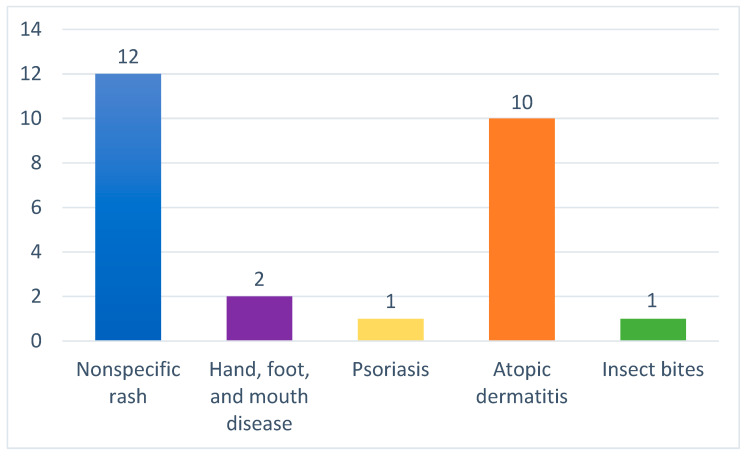
Erroneous diagnoses.

Half of the infants had a previous misdiagnosis, most commonly nonspecific rash (24%) and atopic dermatitis (20%); four infants (8%) had another misdiagnosis (Figure 2).

The mean time to diagnosis was 27 days (SD 9 days), and diagnosis took more than two weeks in more than 45% of cases. According to the IACS consensus [16], the diagnoses were confirmed in 23 cases (45%) (22 by dermoscopy (A3) and only 1 by light microscopy of skin samples) (A1) (Table 3), clinical in 24 cases (47%), and suspected in 4 cases (8%). Most infants (88%) were initially evaluated by their pediatrician; the rest were evaluated in the emergency department. Only 25% were not seen by a dermatologist at any time (Table 4). 

Table 5 shows the different treatments prescribed. All infants but one (98%) received topical permethrin according to the recommended schedule (one cycle of two applications with an interval of seven days between applications); 53% needed more than two cycles. Clinicians prescribed oral ivermectin to 35% of the infants (one cycle of two doses separated by an interval of seven days), and 10% of them needed more than one cycle. Other members of the household received treatment in 98% of cases. Regarding adverse events, 14% of infants had irritant contact dermatitis. The main complication was impetigo, observed in 17 infants (31%), of whom 3 (6%) required hospitalization. Treatment failed in 39 infants (76%).

When we compared the sexes, we found that the girls were more likely to experience pruritus (*p* = 0.04) and have lesions on the scalp (*p* = 0.03).

Infants aged 12 months or older, compared with those aged under 12 months, were more likely to attend daycare, to have excoriation (*p* = 0.02), and to have treatment failure (*p* = 0.02) and were less likely to have lesions on the feet (*p* = 0.03) and the palms/soles (*p* = 0.01).

The probability of living in a household with five or more members was significantly lower among infants of European descent compared with infants of other ethnicities (*p* = 0.01). Infants who lived with four or more people, compared with those who lived with fewer than four people, were more likely to have lesions on the head (*p* = 0.03), hands (*p* = 0.04), palms/soles (*p* = 0.03), and folds (*p* = 0.01) and were more likely to have pustules (*p* = 0.01) and scaly patches (*p* = 0.004). In addition, children who lived in larger family units were more likely to be irritable (*p* = 0.02), to develop impetigo (*p* = 0.02), and to have a time to resolution of more than 30 days (*p* = 0.01).

Time to diagnosis (≤2 weeks versus >2 weeks) was not associated with any epidemiological or clinical factors, but infants with later diagnoses were more likely to have a prior misdiagnosis (*p* = 0.001), mainly nonspecific rash (*p* = 0.001), followed by atopic dermatitis (*p* = 0.07).

Need for additional permethrin cycles was more common in infants with a confirmed scabies diagnosis (compared to those with a clinical diagnosis (*p* = 0.03), those with a previous erroneous diagnosis of nonspecific rash (*p* = 0.08), and those with concomitant use of ivermectin (*p* = 0.02)). These infants mostly belonged to the group that experienced treatment failure (resolution after more than 30 days; *p* < 0.001).

Delayed resolution of scabies (>30 days) was associated with a higher number of household members (*p* = 0.01), diagnosis by a dermatologist (*p* < 0.001), and confirmed scabies diagnosis. These infants were also more likely to have burrows (*p* = 0.03), scaling (0.04), and nodules (*p* = 0.04); to be irritable (*p* = 0.06); and to need more than two permethrin cycles (*p* < 0.001). 

Therapeutic failure was associated with a higher number of household members (*p* = 0.04), diagnosis by a dermatologist (*p* < 0.001), confirmed diagnosis (*p* < 0.001), use of topical corticosteroids (*p* = 0.01) and oral corticosteroids (*p* = 0.05), and need for additional cycles of topical permethrin (*p* < 0.001) and oral ivermectin (*p* = 0.005). 

When we divided the infants into three groups according to time to resolution (<30 days, 30–60 days, >60 days), we found no significant differences between the groups in age or in days to diagnosis, but days to resolution was positively correlated with number of cohabitants (r = 0.28, *p* = 0.04), more cycles of permethrin (r = 0.59, *p* < 0.01), and more cycles of ivermectin (r = 0.47, *p* = 0.05).

## 4. Discussion

Our study confirms that scabies disproportionately affects infants, as almost one-third of the children diagnosed in our hospital were under two years old. The total number of infants in our healthcare area is 5788; therefore, we can estimate that the annual incidence of scabies in infants in the area is 441 per 100,000. Studies have shown that the greater vulnerability to scabies in this age group is due to several factors, such as immunological immaturity [1,2,18] and greater exposure in endemic situations [19], as occurred during the COVID-19 pandemic (although this factor did not affect our population, as data collection began in October 2021).

The mean age of our population was 15 months (SD: 7), with no differences between the younger (≤12 months) and older infants (13–24 months). Among our cases, there were more girls than boys (63% versus 37%); this difference is potentially relevant and may be conditioned by epidemiological factors, although previous studies have found no evidence of this [13].

Most affected infants lived in key points of a specific neighborhood, though this did not constitute an epidemic outbreak. It is an economically disadvantaged neighborhood with small homes and large household sizes. Most neighborhoods affected by scabies share the same characteristics. Social determinants strongly influence diagnostic and therapeutic delay, generate a financial burden for households, and negatively affect health-related quality of life [20].

Daycare is an important source of contagion, since children in this age group have the greatest immunological vulnerability. However, in our study, the main source of contagion was the family (94% of cases), as most of the infants did not attend daycare. The mean number of household members was six (SD: 1), and more than half of the infants lived with four or more people. In addition, 65% of the infants belonged to minority ethnic groups, especially Romani, with high numbers of cohabitants and crowded living conditions that may predispose them to infection [21]. Other predisposing factors (e.g., prematurity, corticosteroid therapy, and asthma) were irrelevant in our case series [22].

The clinical characteristics of the infants studied were similar to those reported in the literature and somewhat different to those of adults affected by scabies [15]: the main lesion types were papules, scaly areas, and excoriations, followed by pustules, burrows, vesicles, and nodules; and the main locations were the trunk, limbs, and head. Our findings thus confirm the particular predilection for acral areas and the scalp in children younger than two years, and we suggest that the presence of lesions on the trunk and arms is related to close and prolonged contact with their caregivers when they hold them in their arms. The fact that more girls had lesions on their heads may be due to more frequent combing or greater use of accessories (hats or bows). Burrows are more common in infants than in adults, probably because of ineffective scratching. More than half of the infants in our series had this lesion type, which confirms it as a pathognomonic feature.

Regarding symptoms, most infants experienced pruritus and irritability. While infants cannot express discomfort verbally, signs of discomfort include crying, exhaustion, daytime sleepiness due to lack of rest, and refusal to eat [23]. Although some of our infants had respiratory symptoms and fever, we did not consider this was due to scabies but rather to the coexistence of a viral infection, which may also have contributed to masking the diagnosis.

The main complication was impetigo, which was more serious and required hospitalization in three infants. 

Twenty-six infants (51%) were given an incorrect diagnosis before the scabies diagnosis. The main misdiagnosis was nonspecific rash, possibly because there were other symptoms pointing to this diagnosis, because the infant did not have typical symptoms of scabies, or simply because the clinician did not consider scabies. The second most common misdiagnosis was atopic dermatitis, which is unsurprising because it is also a generalized pruritic condition that frequently affects infants and can also coexist with scabies.

The mean time to diagnosis was 27 days, and 45% of infants had a diagnostic delay, which was associated with the presence of respiratory symptoms and a previous diagnosis of nonspecific rash or atopic dermatitis (which may have masked the infestation). Our results highlight the complexity of identifying scabies in young children and the importance of considering this condition in differential diagnosis [18]. The criteria established by the ICA facilitate diagnosis, but in parallel with efforts to standardize and improve diagnosis using existing tools, there is a need to develop further diagnostic tests for scabies, especially in small children [19].

We established the diagnosis of scabies according to the international consensus [16], finding confirmed diagnosis in 45% of infants, clinical diagnosis in 47%, and suspected diagnosis in 8%. This indicates a high percentage of confirmed cases.

The infants who were evaluated by a dermatologist (75%) had more severe scabies, which required more cycles of permethrin and took longer to resolve. This suggests that specialized evaluation is performed later, leading to an unfavorable course. Infants who had to wait longer for a diagnosis and who had been misdiagnosed previously received more drugs, had more complications, and were less likely to respond to treatment, suggesting greater severity of the disease.

The scabicide of choice in infants is permethrin [24,25], which was prescribed in 98% of our cases. More than half of the infants needed more than two cycles, which indicates a high rate of therapeutic failure, as discussed by other authors [26]. No infants experienced any serious reactions to the treatment (only eczema in a small percentage), which indicates good tolerance in this age group.

Ivermectin is a second-line treatment for scabies: it can be more toxic in children weighing less than 15 kg and is not approved in that population. However, the off-label use of this drug has extended based on the results of several studies [16,23,26,27,28,29,30,31]. In our series, there were no recorded complications of its use.

The importance of prophylactic treatment for all close contacts coincides with the literature [15,22]. All people who shared a home with the affected infants were systematically provided with prophylactic treatment; however, the high rate of reinfestation suggests that it may not have been used effectively. Nowadays, there is no agreement about the sensitivity of Sarcoptes scabiei to permethrin therapy [32,33]. Inadequate application of the drugs and hygiene measures influence the probability of failure: if the mites and their eggs are not completely eliminated, the infestation may persist, with symptoms returning after a period of apparent improvement. In order to alleviate therapeutic failure in large families, it might be of interest to give precise and detailed instructions in writing to family members on how to apply topical drugs and cleansing fomites. Crowded living conditions and lack of economic resources probably impede full compliance with the hygiene measures.

Therapeutic failure in this study was considerable (76% of the cases) and was associated with a higher number of household members, which may have influenced the spread and persistence of the disease. Similarly, therapeutic failure was associated with the presence of burrows or scaly areas and symptoms such as irritability; this suggests that certain clinical characteristics may predict an unfavorable course of the disease. In addition, diagnostic delay or misdiagnosis that required subsequent confirmation by a dermatologist increased the probability of needing more cycles of treatment and additional treatments, as well as the time to resolution. The role of the dermatologist also extends to monitoring the family and their environment through scheduled follow-up visits [34].

Therapeutic failure with very late healing (60 to 90 days) was not associated with diagnostic delay or other clinical or epidemiological factors except a higher number of household members. These infants received more cycles of permethrin and ivermectin.

Most case series of young children with scabies have been produced in low-income countries and have not analyzed infants specifically [5,18]. Nor have they analyzed factors such as diagnostic delay and lack of treatment response. In Europe, one multicenter study from France analyzed 20 cases of crusted scabies in children under four years [21]; another study compared the response to ivermectin and permethrin in 85 children aged under four years, of whom 60 were aged under two years [19]; and a third study established phenotypic differences in 333 children divided into three age groups, one of which comprised infants [14].

The main limitations of our study are: (1) the small sample size, as it was a single-center study; (2) its retrospective nature, such studies often facing the problem of lack of information in the documentation; (3) the absence of a control group; and (4) the non-availability of official sociodemographic statistical data. 

In conclusion, our series shows a high incidence of scabies in infants. The main source of contagion was the family, and a high proportion of the infants lived in households with five or more members, which was associated with greater risk of contagion, greater therapeutic failure, and clinical characteristics suggestive of more severe scabies.

Diagnostic delay was very frequent and was associated with previous misdiagnosis. There was a high rate of therapeutic failure. It is necessary to establish clinical and social programs to reduce the incidence of scabies in infants, shorten the time to diagnosis, and improve treatment response.

## Figures and Tables

**Table 1:** Epidemiological characteristics.

Characteristics	*n* (%) *
Female sex	32 (63%)
Mean age (months) ± SD	15 ± 7
Age ≥ 12 months	26 (51%)
Mean number of household members	6 ± 1
Risk factors for scabies	
≥5 household members	26 (51%)
Atopic dermatitis	14 (27%)
Prematurity	4 (8%)
Previous corticosteroid therapy	4 (8%)
Ethnicity	
European	18 (35.3%)
Romani	22 (43.2%)
Latin American	7 (13.7%)
Middle Eastern/North African	4 (7.8%)
Source of contagion	
Family	48 (94%)
Daycare	3 (6%)

* Unless otherwise specified in the Characteristics column. *n* = number of infants; SD: standard deviation.

**Table 2:** Clinical characteristics of the infants.

Characteristics	*n* (%)
Type of lesions	
Papules	47 (92%)
Pustules	29 (57%)
Burrows	28 (55%)
Vesicles	24 (47%)
Scaly areas	36 (71%)
Excoriation	38 (75%)
Nodules	18 (35%)
Location	
Head	20 (39%)
Trunk	42 (82%)
Arms	29 (57%)
Folds	23 (45%)
Palms/soles	32 (63%)
Symptoms	
Pruritus	48 (94%)
Irritability	35 (69%)
Respiratory symptoms	8 (16%)
Fever	8 (16%)

*n* = number of infants.

**Table 3:** Summary of the 2020 International Alliance for the Control of Scabies Consensus Criteria for the Diagnosis of Scabies.

**A. Confirmed scabies** At least one of: A1: Mites, eggs, or faces on light microscopy of skin samples A2: Mites, eggs, or faces visualized on an individual using a high-powered imaging device A3: Mite visualized on an individual using dermoscopy **B. Clinical scabies** At least one of: B1: Scabies burrows B2: Typical lesions affecting male genitalia B3: Typical lesions in a typical distribution and two history features **C. Suspected scabies** One of: C1: Typical lesions in a typical distribution and one history feature C2: Atypical lesions or atypical distribution and two history features **History features** H1: Itch H2: Positive contact history

Diagnosis can be made at one of the three levels (A, B or C). A diagnosis of clinical or suspected scabies should only be made if other differential diagnoses are considered less likely than scabies.

**Table 4:** Diagnostic characteristics.

Characteristics	*n* (%) *
Confirmed diagnosis	23 (45%)
Clinical diagnosis	24 (47%)
Suspected diagnosis	4 (8%)
Mean time to diagnosis (days) ± SD	27 ± 9
Diagnosis ≤ 2 weeks	28 (55%)
Diagnosis > 2 weeks	23 (45%)
Evaluation by dermatologist	38 (75%)

* Unless otherwise specified in the Characteristics column. *n* = number of infants; SD: standard deviation.

**Table 5:** Treatment received.

Treatment	*n* (%)
Scabicides	
Permethrin	50 (98%)
Permethrin (>2 cycles)	1 (2%)
Ivermectin	18 (35%)
Ivermectin (>1 cycle)	5 (10%)
Benzyl benzoate	8 (16%)
Sulphur	8 (16%)
Other treatments	
Oral antihistamines	38 (75%)
Topical corticosteroids	29 (57%)
Oral corticosteroids	10 (20%)
Topical antibiotics	23 (45%)
Oral antibiotics	12 (24%)

*n* = number of infants.

## Data Availability

The datasets presented in this article are not readily available because they are part of a larger study still in progress. Any additional information can be requested from the main author.
